# Vision through Healthy Aging Eyes

**DOI:** 10.3390/vision5040046

**Published:** 2021-09-30

**Authors:** Nir Erdinest, Naomi London, Itay Lavy, Yair Morad, Nadav Levinger

**Affiliations:** 1 Department of Ophthalmology, Hadassah-Hebrew University Medical Center, Jerusalem 91120, Israel; nir.erdinest@mail.huji.ac.il (N.E.); itayl@hadassah.org.il (I.L.); nadav.levinger@gmail.com (N.L.); 2 Even Israel 5, Jerusalem 94228, Israel; 3 Assaf Harofeh Medical Center, Department of Ophthalmology, Zerifin 70300, Israel; yair.morad@gmail.com; 4 Enaim Refractive Surgery Center, Department of Ophthalmology, Jerusalem 94383, Israel

**Keywords:** aging, vision, visual acuity, visual fields, contrast sensitivity, dry eye

## Abstract

As life expectancy grows, so too will the number of people adversely affected by age. Although it is acknowledged that many conditions and diseases are associated with age, this mini-review will present a current update of the various visual changes that generally occur in healthy individuals disregarding the possible effects of illness. These alterations influence how the world is perceived and in turn can affect efficiency or the ability to perform ordinary daily tasks such as driving or reading. The most common physical developments include a decreased pupil size and retinal luminance as well as changes both in intercellular and intracellular connections within the retina along the pathway to the visual cortex and within the visual cortex. The quantity and the physical location of retinal cells including photoreceptors, ganglion and bipolar retinal cells are modified. The clarity of intraocular organs, such as the intraocular lens, decreases. These all result in common visual manifestations that include reduced visual acuity, dry eyes, motility changes, a contraction of the visual field, presbyopia, reduced contrast sensitivity, slow dark adaptation, recovery from glare, variation in color vision and a decreased visual processing speed. Highlighting these prevalent issues as well as current and possible future innovations will assist providers to formulate treatments and thereby conserve maximum independence and mobility in the modern mature population.

## 1. Introduction

Many significant visual changes can occur as humans mature. The predominant visual changes include a decreased visual acuity and binocular function, dry eyes, ocular motility changes, a contraction of the visual fields, presbyopia, a decreased contrast sensitivity and dark adaptation, delayed glare recovery, changes in color vision and a decreased visual processing speed [1,2,3]. The most prevalent physical developments include a decrease in the pupil size and retinal luminance as well as changes both in intercellular and intracellular connections within the retina along the pathway to the cortex and within the visual cortex [4,5,6,7,8]. Alterations also occur in both the quantity and physical location of various cells such as photoreceptors, ganglion and bipolar retinal cells as well as a decrease in the clarity of intraocular organs—as happens, for example, in the intraocular lens [1,3]. People are invariably affected differently due to genetics along with environmental factors such as diet and physical and mental activities. A decline in visual abilities potentially influences the quality of life and may impact on the performance of common everyday tasks such as reading, computer work, driving, maneuvering through a crowded or unfamiliar environment, locating desired objects among clutter and even walking, which potentially causes falling [3,9].

**Figure 1:**
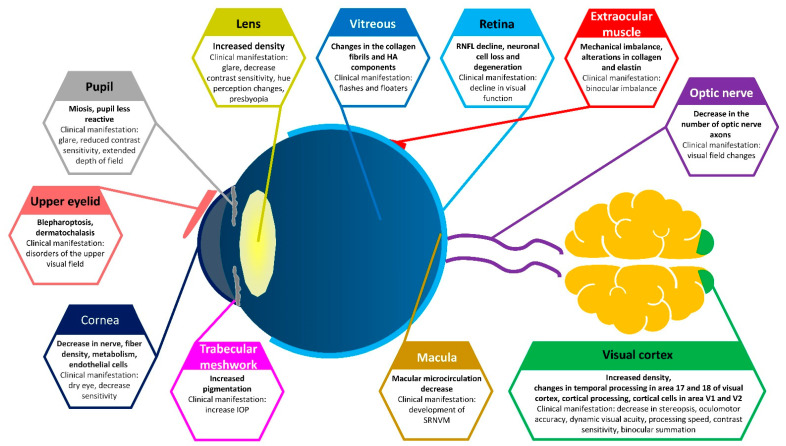
Primary physiological changes during aging and their clinical manifestations. SRNVM: subretinal neovascular membrane; IOP: intraocular pressure; HA: hyaluronic acid.

A few processes cannot be interrupted but several developments can be reversed or at least slowed using early prevention with a single process or a combination of external resources such as optical devices, pharmacological treatments or training. Recognizing the hypothesis that the visual system is both affected and affects other systems in the body, the objective in the following review is not to provide a comprehensive overview of the entire field of vision and aging but to consider the most common visually related changes that occur in healthy individuals, disregarding the effects of ocular diseases even if those diseases are associated with age. The contemporary and developing mature population engages in highly demanding modern-day activities, such as computer and cellphone use, and expects to maintain their active lifestyles as long as possible. Identifying the various age-related impairments, the mechanisms underlying these changes and how they impact current everyday activities can help caregivers appropriately address and guide their patients to preserve maximum independence and mobility (Figure 1).

## 2. Visual Acuity

When discussing visual acuity, we need to differentiate between observing static objects versus dynamic visual acuity, which is observing moving objects. In the past, research had shown that a deterioration in static visual acuity began from an age as young 50 but newer studies have revealed that visual acuity generally remains relatively stable at least until the age of 65 [2,8]. It is important to mention that these newer studies finding that acuity endures until a later age addressed and eliminated the effects of glare and low contrast [2,10]. It was further noted that visual acuity was affected by the number of digits per line, how crowded the rows were and whether the subject had a time limit to respond [11,12]. It has been suggested that a reduced acuity at a younger age may possibly be partially a result of these aforementioned interferences not been acknowledged and removed [13,14,15]. As the density of the cone photoreceptors, which are responsible for visual acuity, is relatively stable throughout life, it is postulated that the decrease is connected with the change in the number of retinal ganglion cells and bipolar cells or the connections between them [6,7,8,16,17,18,19]. Binocular visual summation is the acuity when both eyes are open and it is almost always superior to monocular acuity [20,21]. This appears to be a result of cortical processing of the information collected from the two eyes. Binocular summation advantage (or lack thereof) is more apparent when testing in a low contrast environment as opposed to high contrast situations [20,21]. An age-related decline, occurring noticeably at around age 40 and then at around age 60, is attributed to neuronal cell loss and increased neural variability, most likely higher than the primary visual cortex (which is known as area V1) [22,23,24,25,26].

Dynamic visual acuity is the ability to detect objects when they are in motion. This becomes increasingly difficult as people age, more so under low illumination or high glare conditions [27,28,29]. Researchers are attempting to quantify this decline, but as there is no accepted method of evaluating or units to measure this type of acuity, it is difficult to give a specific value and properly express the deterioration [30,31]. Multiple components affect the perception of motion and several have been shown to be more affected by age such as a higher threshold of the minimum contrast and speed needed to determine the direction of motion as well as the surround suppression of motion. Discriminating a desired target weakens in the central vision (translating to more difficulty in segregating moving objects in the periphery from their backgrounds as opposed to an object in the central vision) [30,31]. A deteriorated visual input caused by decreased visual acuity can also influence the efficacy of the vestibule-ocular reflex and, consequently, posture and balance [2]. Vernier acuity describes the ability to accurately align two dots or lines horizontally or vertically. This ability usually remains stable throughout life when the targets are sharp and other optical disturbances are neutralized [32,33,34,35].

## 3. Dry Eye

Dry eye is one of the most common ocular problems and affects approximately half the population with or without symptoms [36,37,38]. The prevalence rises and exacerbates as people age, especially in women, due to hormonal influences [36,37,38]. As fluctuating or blurry vision is one of the most prevailing symptoms of dry eye, appropriate diagnosis and management are imperative. Other symptoms include general discomfort, burning, pain, foreign body sensation, photosensitivity and tearing [39,40]. Both the rapid evaporation of the tears or an insufficient production can be the cause [39,40].

A study was specifically designed to investigate the changes in tear stability and compared both biochemical and physical parameters in two cohorts separated in age by over 30 years, the older group 63.0 ± 4.0 years. A few correlations between the clinical and biochemical responses were found such as corneal staining and bulbar conjunctival redness, which coincided with elevated albumin (a biomarker for vascular permeability) and a decrease in lysozyme, lipocalin and lactoferrin proteins (the primary aqueous secretory tear proteins), which is often clinically expressed as a reduction in the tear flow rate and tear break-up time [41].

Substance P and calcitonin gene-related peptide (CGRP) are neurotrophic mediators that are released from corneal nerve endings and play an important role in corneal epithelial wound healing [42,43]. The number of corneal nerves decreases with age and with it comes a decrease in these mediators in the tears [43]. A delayed inflammatory response can result as well as decreased tear production, especially reflex tears. With less tear volume, there is less tear turnover, which can raise tear osmolality [42,43,44,45,46]. Elevated osmolality irritates and can damage the epithelial cells as well as stimulate the nerve cells on the surface of the eye [44,45,46]. This in turn can stimulate the release of more inflammatory mediators and stress signals and further raise osmolality [47,48,49]. Elevated osmolality with age is occasionally attributed more to females; less of a correlation with age has been found in males [50,51]. Inconsistent findings between studies have been reported when discussing a decreased lipid volume and tear viscosity [52,53,54,55]. A decrease in active meibomian glands resulting from an atrophy of the meibomian gland acini, cystic dilation and focal hyperkeratinization of the ductal epithelium and secretion can be attributed to age, which directly affects evaporation and osmolality [51,52,53,56,57,58]. Alternatively, many consider behavior and external causes such as incomplete blinking, which can be an independent occurrence or stimulated by digital screen use, to be a source of meibomian dysfunction rather than age [59].

An additional physiological parameter to consider is the decrease in the endothelial cell quantity at a rate of approximately 0.5–0.6% per year [60,61], slightly more in the peripheral cornea [62]. The primary function of this layer is to maintain corneal clarity through its pump action. Endothelial loss has been found to be correlated with severe dry eye, hypothesized to be attributed to reduced corneal nerves present both in the healthy elderly and in patients with dry eye disease [63]. If the cell decline both in number and functional capability is substantial, the corneal thickness potentially increases causing suboptimal corneal transparency [64,65]. Symptoms can include a decreased acuity and contract sensitivity or increased glare [66,67,68].

The majority consensus in the literature suggests that the basal tear secretion rate stays relatively constant throughout life [69,70,71,72]. As far as an explanation regarding those that elude dry eye signs and symptoms, one theory suggests tear physiology; the stability, volume, evaporation, lipid layer structure and osmolality all remain fairly constant over time or decline at a rate that does not compromise the function. An alternative theory suggests that these parameters may decline but the reduced rate of drainage preserves the equilibrium [73,74].

Clinical signs of dry eye include corneal staining, a decreased tear prism and tear break-up time, meibomian gland dysfunction and varying degrees in severity of blepharitis. Treatments vary depending on both the patient discomfort and clinical presentation and range from environmental changes such as ambient humidity and blinking exercises, topical artificial tears or prescription ointments as a palliative treatment to preserve corneal integrity to dietary modifications such as an omega-3 or a cyclosporin A intake to lower the inflammatory response or external treatments such as heat compresses and intense pulse light or radio frequency treatment to improve the meibomian gland function [75,76,77,78,79].

## 4. Binocular Vision

There is a decrease in binocular vision as people age, divided into mechanical and perceptual sources [20,30]. One hypothesis as to a perceptual cause is a reduction in the visual cortical processing [20,21,30]. Changes in collagen and elastin in the body mechanically modify the tone of the extraocular muscles. As a result, the pulling versus the relaxing relationship between the muscles changes, which in turn alters the eye position and the balance of power between the ocular muscles to stabilize the eye in various positions of gaze [80]. Although looking downward seems to be largely unaffected, a limited angle of upward gaze seems to be a commonly impaired direction of gaze [80,81].

Binocular input is what provides a precise depth perception and allows for accurate spatial awareness and navigation [2]. True binocular vision requires ultimate coordination, harmony and correspondence between the two eyes. A slight slippage between the two eyes is occasionally enough to turn a phoria into a tropia [2,20,21,30]. Common manifestations of an imbalance are a divergence insufficiency, a convergence insufficiency or sagging eye syndrome, which can conceivably occur at every age but become much more prevalent after an age of 70 [2,20,21,30]. Treatment provided is decided on an individual basis and can be either optical or training. After the age of 70 there is often a decrease in stereo acuity, which is also attributed to a reduction in neurons in the striate cortex in the visual cortex of the brain [20,21,30,82]. It is difficult to separate stereo acuity from visual acuity completely as it is significantly affected and reduced if each eye does not see well enough, stressing the importance of correcting refractive errors in order to achieve maximum stereo acuity [83,84]. Most research was conducted under binocular conditions so it is important to consider the possibility that the results were affected by monocular blur [20,21,30,82].

## 5. Ocular Motility

A moving object of interest is accurately tracked using a combination of fixation pursuits and saccades. These complex processes must also be capable of adapting to modifications that develop throughout life affecting the forces acting on each eye. Examples of alterations include displaced extraocular muscles, changes in orbital fat, a decrease in the quantity of muscle fiber and sensitivity of the muscle spindles [81]. As people age, the aperture also increases and the eye sockets become wider. Many studies have found that age brings a difficulty both at maintaining fixation as well as suppressing unwanted eye movements [80,85,86,87]. Smooth pursuits are often interrupted with catch-up saccades [81,88]. One must recognize that visual tracking and reaction time are attention sensitive, which is independently adversely affected as people age [89]. Numerous elements of the saccade movement are affected including an increase in the direction error rate (although there are disagreements between studies) and a variability in accuracy specifically in anti-saccades but it is generally preserved in pro-saccades and reaction times [80,88]. The skills required are far more complex and beyond the focus of this review. Briefly, one must consider not only accurate saccade movements but also perceptual span (the area of the text or how many letters a fixation encompasses) and where in a word the individual lands each saccade (at the beginning, center or end of the word) [89,90]. Research indicates a possible increased difficulty in using parafoveal information in older adults as well as noted longer fixations and more regressions, a result of skipping, which occurs due to inaccurate saccades [85,89]. These changes can manifest both when navigating through space as well as when trying to read. Eye movement training has been shown to have a significant positive effect in post-stroke patients and a voluntary saccade protocol has been suggested for both patients with Parkinson’s disease and healthy adults [81,91].

## 6. Visual Field

Determining the peripheral field is threefold: seeing the object, the localization of an object in space and the speed of movement of the object [85,89]. There is a general constriction of functional visual fields and peripheral vision especially when the surroundings demand divided attention [85,89]. The loss is occasionally defined as an absolute scotoma and occasionally as a functional loss. It is as yet unclear why a few people are able to compensate for such a field loss with eye movements and a few are not [2,4,9,31,92].

A shrinkage of several degrees of the peripheral vision in each decade above the age of 45 has been observed and is greater above the age of 65 [93,94]. The affected size of the visual field depends on the size of the observed target, luminance, color (notably blue) and the contrast between the target and the background [95]. After the age of 60, a decrease in sensitivity to dark adaptation, flicker and perimetry field tests has been observed [93,94]. The source of these changes is believed to occur in the retina and the changes are associated with changes in retinal metabolism, a result of a possible decrease in oxygen tension [96]. One study found the mean rate of loss of sensitivity between the ages of 20 and 40 was 0.101 dB per year and between the ages of 40 and 60, the loss was 0.172 dB per year [97].

Dermatochalasis, which potentially progresses as people age, can cause a physical obstruction on the upper visual field and can be corrected surgically. Accurately assessing the object location in the periphery deteriorates with age but generally people are able to gage the speed of an object throughout life [2,4,9,31,92].

## 7. Presbyopia

Various photochemical changes occur to the intraocular lens as well as modifications to cytoplasm in the fibers in the lens cortex and to the solubility of protein in the nucleus [1,3]. Consequently, the elasticity of the cortex changes and the nucleus become stiffer and denser, resulting in an inability to increase the anterior convex curvature that would bring the focal point closer as required when observing a near target [1,98]. This process is termed presbyopia and begins to affect functionality approximately around the age of 40 [1,3]. Initially, the symptoms are eye discomfort or a distance blur for a few seconds after extended near work, which ultimately turns into blurry near vision [1]. Although a prevalent adaptation is to just hold the material further from the eyes, an external solution usually becomes unavoidable beyond the age of 50 and is customarily composed of an optical aid such as spectacles or contact lenses, which generally restore full visual function [1]. Pharmacological treatments are in various stages of development, which target either the parasympathetic system to initiate miosis and extend the depth of the field or the intraocular lens in an attempt to soften the crosslinked proteins as well as surgical innovations including intraocular implants based on multiple philosophies (such as accommodation or an extended depth of focus) [3,99,100,101].

## 8. Contrast Sensitivity

Contrast sensitivity is the disparity of light between an object and its background or between two adjacent objects [102]. Deterioration varies in photopic versus mesopic or scotopic environments [102]. It seems to be related to optical changes such as pupil miosis, a decrease in the concentration of photoreceptors (especially in the periphery) and to neurological causes including a reduction in the quantity and efficiency of neurons [103,104,105,106]. Specifically, this was found in the magnocellular pathway, which begins from the retinal ganglion cells and progresses to the magnocellular layers at the lateral geniculate nucleus (LGN) on to the primary visual cortex [107,108].

In a well-lit environment, studies have found that contrast sensitivity was affected predominantly when patients were presented with mid- to high-frequency targets [10,107,109]. Contrast sensitivity decreased primarily at low frequencies in low illumination to the extent that researchers determined this to be a more significant cause for motor vehicle accidents than a loss of visual acuity [10,107,109]. In addition to the abovementioned, the decline is caused by an increased intraocular lens density, internal reflections and light scatter and a general decrease in retinal illumination [10,107,109,110]. Studies found that a decreased sensitivity manifesting as a difficulty in identifying faces supported the physiological change with finding changes in the visual cortex [10,109,110,111].

## 9. Dark Adaptation

The rapid regeneration of rhodopsin in photoreceptors is essential for dark adaptation [112]. Changes in the retina over time include a thickening of the retinal layers, specifically Bruch’s membrane. Additionally, a build-up of extracellular material between the layers and alterations in the retinal pigment epithelium occur. This build-up causes a localized deficiency of vitamin A, which leads directly to a rise in the adaptation threshold (and a delay in adaptation to low illumination) [112,113,114]. In addition, there is a mid-peripheral loss of rods at a rate of 0.4% per year. On average, an elderly person can take ten minutes to adjust to an environment that a young person would adapt to in two minutes; a significant delay [112,113,114]. A simple aid is to suggest the use of dark lenses encompassing the visual field in a very bright environment, thereby decreasing the adaptation time as light bleaching is avoided.

## 10. Glare Recovery

Studies have found a linear increase in visual acuity loss when exposed to the same glare source as people age, which then rises exponentially after the age of 65 [115,116,117]. In practical terms, it was found that a young person would lose up to one line of visual acuity (or less) whereas older people could lose up to five lines of visual acuity when exposed to the same source [117,118,119]. After a minimal exposure to a glare source, the recovery to best visual acuity can take up to three minutes longer in an elderly person compared with a young person. Additional studies found it can even take nine times as long to recover to the initial visual acuity after an exposure to a glare source. Glare can affect visual quality with respect to spatial orientation, contrast and color, even in photopic settings [117,118,119].

## 11. Color Vision

Aging influences color perception even when other characteristics such as visual acuity are not at all impaired [120]. Throughout the aging process, the intraocular lens absorbs more and more of the short wavelengths as a result of an accumulation of yellow pigment, which results in a slight blue-blindness [17,108]. In addition, light is scattered more in the intraocular media with age, mostly because of the intraocular lens. This in turn affects the sensitivity of all three-color photoreceptors [11,16,17]. The amount of light reaching the retina is further affected by pupil miosis and changes in the media. The direction of the rods becomes less perpendicular and their reduced numbers cause less photons to be absorbed in the photoreceptors or they are absorbed on an angle resulting in a less saturated color that is occasionally even perceived as a different color. The perception of a different color when light is absorbed on an angle, otherwise known as the Stiles–Crawford effect, occurs throughout life in the receptors out in the peripheral retina but here it manifests more centrally [11,16,17,121]. The difficulty is more at differentiating between similar hues (especially in the short to medium wavelength colors such as blue versus green) rather than between completely different colors (red versus blue) and is affected more in low illuminated surroundings [8,11,16,17].

## 12. Speed of Visual Processing

This skill can be defined as the amount of time required to make a judgment about a visual stimulus. It can include many types of visual tasks such as detecting the presence of or identifying a target, discriminating between targets, recognizing the target is familiar and indicating its location in space as well as other decisions about visual events. There is a slowing in the ability to process visual information with age, particularly in object identification, location and differentiation from a background [9,92,122]. The loss is increased when there is a crowding of objects in the visual field or tasks that demand divided attention. These studies included healthy subjects and eliminated people with neurological problems such as cases of Alzheimer’s or dementia, showing as well that the phenomenon is not connected to cognitive issues such as short-term memory loss [122]. It is further differentiated from neurological changes such as navigational impairment, which has been connected to deficits in the ability to mentally represent space in an object-centered (allocentric) format rather than in a self-centered (egocentric) format [123], and can definitely affect activities such as driving ability [9,124,125,126,127]. The speed of processing can be improved with training that focuses on both improving the speed of target searching and the ability to perform one or more attention-demanding task quickly [128,129,130].

## 13. Developing Modes of Intervention and Prevention

Although not specific to the ocular system, alterations in the cellular pathways and changes in cell genes, both at the protein and post-translational level as cells age, lead to a decline in function and occasionally can progress to disease. Investigating the causes and examining common patterns of changes as well as identifying biomarkers are part of the challenging tasks for researchers in their effort to create effective therapeutics.

Current interventions include genetic, pharmacological and non-pharmacological approaches or lifestyle modifications such as diet to control both nutrient intake as well as metabolic regulation and exercise [131].

Tele-health has expanded to the extent that it has become part of routine care in parts of the world [81]. Oculometrics pertains to the measurements of various eye movements including, but not exclusively, the oculomotor function, pupillary responses and optokinetic responses, a few of which, as mentioned, alter with age. Research is still endeavoring to create reliable and accessible systems to remotely monitor data such as eye-tracking. As the cortical and subcortical areas of the brain are involved with ocular movements, tracking or monitoring these movements potentially provides an indirect window to the neural and cognitive processing status [81].

The utilization of artificial intelligence (AI) in healthcare and medicine is accelerating. As molecular, biochemical and genomic data of both healthy and unhealthy individuals are amassed, AI uses this information to identify biomarkers or features specific to certain processes and can then establish targets to treat. Ultimately, data can be used to innovate drugs or therapy and screening techniques can streamline a patient to a proper diagnosis and eventually may even be capable of optimizing the treatment for each individual [131].

## 14. Summary

The changes to the visual system that occur in the aging population affect daily activities in modern-day life and the quality of life of an ever-greater population. The predominant changes include many aspects of the visual sense including visual acuity, binocularity, contrast sensitivity, adaptation to changes in illumination, glare and visual processing. The environment, life choices and genetics all influence who will be affected, at what pace and to what capacity. As the decline occurs, the modes of treatment vary from optical to pharmacological to physical rehabilitation. Governments or health ministries can help to educate caregivers and the public and perhaps guide referrals or co-management with eye care practitioners who are experienced in working with this population to maximize care. Research continues to strive to decipher and ultimately control the plethora of underlying processes and to provide solutions to changes that affect modern everyday living so profoundly.

## Data Availability

Not Applicable.
